# Non-invasive methods to measure inter-renal function in aquatic salamanders—correlating fecal corticosterone to the environmental and physiologic conditions of captive *Necturus*

**DOI:** 10.1093/conphys/coz074

**Published:** 2019-11-11

**Authors:** Andrew H Nagel, Mark Beshel, Christopher J DeChant, Sarah M Huskisson, Mark K Campbell, Monica A Stoops

**Affiliations:** 1 Center for Conservation and Research of Endangered Wildlife, Cincinnati Zoo & Botanical Garden, 3400 Vine Street, Cincinnati, OH 45220, USA; 2 Jacksonville Zoo and Gardens, 370 Zoo Parkway, Jacksonville, FL 32218, USA; 3 Innovative Zoological Solutions, 816 Suire Ave, Cincinnati, OH 45205, USA; 4 Omaha’s Henry Doorly Zoo and Aquarium, 3701 South 10th Street, Omaha, NE 68107, USA

**Keywords:** ACTH, amphibian, glucocorticoids, reproduction, waterborne hormone

## Abstract

This study sought to develop non-invasive techniques to monitor glucocorticoids in captive *Necturus* as a means to correlate inter-renal gland function in relation to environmental and physiological changes. Six individually housed breeding pairs of captive *Necturus beyeri* were subjected to seasonal changes in water temperature (30°F temperature differential) to stimulate natural breeding, specifically spermatophore deposition and oviposition. An enzyme immunoassay was validated for the measurement of *N. beyeri* faecal corticosterone metabolites (*f*CMs) by exhibiting parallelism and accuracy to the standard curve. Longitudinal (December 2016—October 2017) assessment of *f*CM concentrations and pattern of excretion from samples collected from the six breeding pairs revealed a seasonal inter-renal effect with higher concentrations (*P* < 0.05) excreted during months (December–March) of the year associated with breeding activity and when water temperatures were lowest. Males from each pair produced spermatophores starting on 08 December 8 2016 and ending on 05 April 2017. Females from four of the six pairs went on to successfully oviposit eggs in mid-late April 2017. One clutch was fertile, and three were non-fertile. No differences (*P* > 0.05) were detected in *f*CM concentrations between pairs in which oviposition did or did not occur. In addition, a novel waterborne corticosterone metabolite (*w*CM) assay was validated to overcome challenges associated with faecal collection in a group-housed amphibian. An adrenocorticotropic hormone (ACTH) challenge performed in an adult male *Necturus maculosus* resulted in a 50-fold increase in *w*CM at 4 h post-injection and marked the first demonstration of a waterborne inter-renal response to ACTH in *Necturus*. This study not only provides insight into inter-renal function in an aquatic salamander that exhibits marked reproductive seasonality but also confirms utility of *f*CM and *w*CM measurements as non-invasive means of assessment.

## Teaser

We measured faecal corticosterone metabolites (*f*CMs) in aquatic salamander *Necturus* to understand how inter-renal function changes throughout seasonal environmental changes and associated reproductive activity. *f*CMs elevated with lower water temperatures and breeding activity. To overcome some challenges associated with faecal hormone collection/analysis, waterborne corticosterone (*w*CM) was validated through ACTH challenge.

## Introduction

As the biological integrity of freshwater ecosystems continue to decline in extent and quality across the globe, threats to biodiversity intensify. Many of the earth’s amphibians are being lost on an exponential scale. Most amphibian species rely on an aquatic environment to support specific life stages, but those exhibiting neoteny spend their entire lifespan in water. Several species of fully aquatic salamanders inhabit freshwater ecosystems throughout North America. The *Necturus* genus consists of five currently recognized species (*N. maculosus* (LC), *N. beyeri* (LC), *N. lewisi* (NT), *N. punctatus* (LC) and *N. alabamensis* (EN)) with two listed as Near Threatened or Endangered by the International Union for Conservation of Nature (iucn.org, downloaded 19 May 2019). As they do not undergo the metamorphosis which terrestrial salamanders do, they may be more susceptible to factors such as chytrid fungus, climate change and pollution, making it ever more difficult for aquatic ecosystems to support their populations in abundance (Chatfield *et al*., 2012). Given the continuing pressure on many local *Necturus* populations, captive propagation programs are imperative to safeguard species survival (Stoops *et al*., 2014).

The biggest obstacles faced in establishing successful captive breeding programs centre around health and reproduction. Breeding in most amphibians occurs in a seasonal manner (Bertoluci and Rodrigues 2002, Semlitsch and Pechmann 1985). Mimicking the intricate environmental conditions found in nature that act to synchronize seasonal reproductive responses can be difficult to achieve in captive situations. Additionally, it appears many species require intricate courtship displays to promote successful reproduction (Houck and Arnold 2003, Treer 2013). Captive female amphibians frequently fail to produce eggs, and if they do, ovulation and oviposition are not guaranteed. Their male counterparts are often unsuccessful in producing or releasing spermatozoa/spermatophores. Recent advances in the application of assisted reproductive techniques (ARTs) to captive amphibian populations have been used to help overcome some of these challenges, most notably for *Anurans* (Calatayud et al., 2018, Kouba and Vance, 2009). *Necturus* are unique in that they exhibit the reproductive strategy of internal fertilization, with spermatophore deposition preceding oviposition by several months. Establishing physiologic measures associated with normative reproductive processes would help in determining when intrinsic and/or extrinsic manipulations positively or negatively impact *Necturus* reproductive potential and output in a managed setting.

Endocrine studies involving amphibians frequently rely on the use of plasma or tissue samples that can be limited to a single measure and interpretation confounded by the physical restraint needed to obtain the biological material. Non-invasive faecal, urine and salivary hormone monitoring have been reported in several anuran species (Germano *et al*., 2009, Hammond *et al*., 2018, Hogan *et al*., 2013, Kindermann *et al*., 2012, Narayan *et al*., 2010a, 2010b, 2011, 2016, Szymanski *et al*., 2006), but have not yet been reported in any species of salamander. It can be logistically and physiologically infeasible to collect daily samples from some species, and, as many amphibians are group-housed, determining which sample came from which individual can be challenging. Dermal secretions have recently been shown as a novel matrix for monitoring glucocorticoid concentrations in a diverse array of amphibians (Santymire *et al*., 2018, Scheun *et al*., 2019). The utility of secreted hormones in water have similarly been established in several small fish and amphibian species (Ellis *et al*., 2004, Gabor *et al*., 2013, 2016, 2018, Kidd *et al*., 2010, Reedy *et al*., 2014) but have not yet to be applied to larger-sized amphibians. Corticosterone, the primary glucocorticoid secreted by amphibians, reptiles and birds, aids in the regulation of metabolic, immune, behavioural and reproductive function (Crespi *et al*., 2013, Moore and Jessop, 2003). Although a chronic increase in corticosterone can have numerous detrimental effects, such as suppressing immune function, gonadal hormone production and somatic growth, the effect thereof is generally linked to season and/or specific life stages (Crespi and Denver, 2005, Crespi *et al*., 2013, Dayger and Lutterschmidt, 2017, Moore and Jessop, 2003, Narayan 2013). With its intricate role in numerous body systems, glucocorticoids exist as important biomarkers in determining changes in inter-renal function throughout major life history events such as reproduction. Changes that facilitate behavioural and physiological aspects of reproduction are especially important to understand so as to apply to captive propagation efforts for amphibians.

The purpose of this study was to validate non-invasive measures of inter-renal function in *Necturus* via enzyme immunoassay (EIA) of corticosterone. Specifically, longitudinal measurements of faecal corticosterone metabolite (*f*CM) concentrations and patterns of excretion were conducted in multiple natural breeding pairs of *N. beyeri* in captivity. As *Necturus* exhibit defined reproductive seasonality (Shoop 1965, Stoops *et al*., 2014), we hypothesized that elevated *f*CMs would be measured during the breeding season to meet the changing energetic demands associated with reproduction. Observations of spermatophore deposition and oviposition were noted. In addition, we determined if a novel waterborne hormone analysis technique developed for smaller amphibians could be adapted for measurement of corticosterone from large fully aquatic salamanders. An adrenocorticotropic hormone (ACTH) challenge was conducted in *N. maculosus* with excretory concentrations and lag time of waterborne corticosterone metabolites (*w*CMs) compared between anaesthetic events associated with administration of ACTH or a sterile water control.

## Materials and methods

### Animals

Procedures were approved by the Cincinnati Zoo and Botanical Garden (CZBG, 15-128) and Jacksonville Zoo and Gardens (JZG) Institutional Animal Care and Use Committees. Faecal collection and hormone analysis were conducted from December 2016 through October 2017 on six *N. beyeri* breeding pairs at JZG. All animals were sexually mature having entered captivity from the wild in 2008 as juveniles. Each pair was housed in 22-gallon containers with flow-through design (i.e. inflow on one end and outflow on the other end via standpipe). Each container held ~10 gallons of water at any time. Pea gravel was used as substrate, and 8″–10″ clay saucers served as hide/oviposition sites. Water flowed into a sump through 100- and 10-micron filters, respectively, with UV sterilization applied to water before returning to the container. Diet consisted of live red wigglers (*Eisenia fetida*), European nightcrawlers (*Eisenia hortensis*), Canadian nightcrawlers (*Lumbricus terrestris*) and captive-reared mosquitofish (*Gambusia affinis*). Water temperature (Aqua Logic Inc., digital temperature controller) and light cycle varied throughout the year to simulate wild conditions (Shoop, 1965, Stoops *et al*., 2014). Specifically, at the beginning of October water temperature declined in 2°F increments each week from a seasonal high of 74°F to a seasonal low of 44°F achieved on 08 January 2017. This temperature (44°F) was maintained for 52 days before being incrementally increased on the first week of March 2017. Each tank reached the seasonal high water temperature (74°F) by 11 June 2017 and stayed there for 112 days until the seasonal decline was initiated during the first week of October 2017. Dates of faecal collection, spermatophore deposition and oviposition were recorded. A single adult non-breeding male *N. maculosus* (95 mm snout–vent length, SVL) at CZBG maintained in a 30-gallon glass tank with constant 70°F water and exposed to natural room lighting was subject to waterborne hormone analysis.

### Faecal hormone collection and extraction

Faecal samples were collected using individual pipettes, transferred to 1.5-mL Eppendorf tubes, labelled with tank ID and date and stored at −20°C. Frozen samples were shipped on dry ice from JZG to the CZBG endocrine lab where they were freeze-dried (Benchtop Freeze Dry system, VirTis Warminster, PA), weighed to ≥0.05 g into 15-mL polypropylene conical tubes (USA Scientific, Ocala Florida), extracted with 80% MEOH at a dilution factor of 20 and rotated overnight (Kummrow *et al*., 2011). After rotation, samples were centrifuged (2800 *g*, 15 min), and the supernatant was removed and stored in 2-mL cryovials at −20°C before analysis.

### Waterborne hormone collection and ACTH challenge

**Figure 1:**
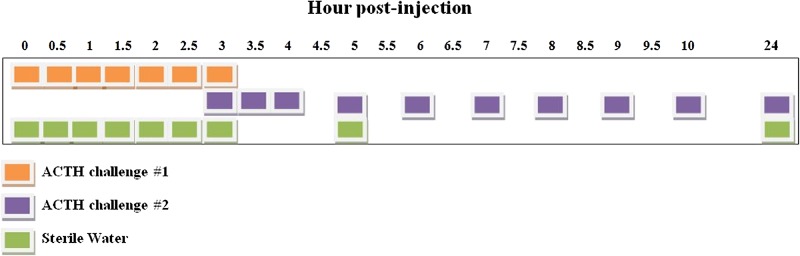
Collection schedule for waterborne corticosterone metabolite (*w*CM) measurement in captive *Necturus maculosus* following anesthetic events associated with ACTH (*n* = 2) or sterile water (*n* = 1) administration

Nitrile gloves were worn throughout all waterborne collection processes. Three separate anaesthetic procedures were conducted (Calatayud *et al*., 2019, Stoops *et al*., 2014) on *N. maculosus* at 3- to 5-week intervals; two procedures were associated with a single i.p. injection (27G, 125 μL) of 10.7 IU ACTH (A6303; Sigma-Aldrich, St. Louis, MO, USA) dissolved in sterile water, and one control procedure consisted of i.p. injection of 125 μL sterile water only. Before anesthetic procedures, 1 L volumes of adjusted RO water (0.2 g MgSO_4_ per liter) were prepared for each time point that waterborne hormone analysis was collected post-injection (Fig. 1). Injections were performed between 08:00 and 10:00 to not confound any circadian cycle in inter-renal activity. Following full anaesthetic recovery, the animal was moved to a glass 2.5-gallon tank (12′ × 6′ × 8′) filled with 1 L adjusted RO water, where it remained undisturbed for 30 min, for the collection of secreted/excreted hormones. For each collection time, the animal was moved to a new tank containing fresh 1 L adjusted RO water. Glass tanks used for collection were rinsed thoroughly with hot tap water three times and dried before reuse. Collection of baseline *w*CM concentrations was conducted when the specimen was not subjected to anaesthesia. The animal was removed from its tank at 09:00 and immediately transferred to 1 L adjusted RO water where it remained for the 30-min collection period.

### Waterborne hormone extraction

Water samples were filtered (Whatman®, 11 μm pore size) using Supelclean LC-18 Solid Phase Extraction columns (Sigma-Aldrich., 505471) connected to a custom manifold. Cap adapters (Sigma Aldrich, 57 020-U) were placed on top of each column, and Tygon tubing (R-3603) connected the cap to each 1-L sample. The manifold consisted of a 2-L glass filter flask fitted with a size 6 rubber cork that had four vertically driven 14G needles fitted into it for the columns to attach. Columns were primed twice with 2 mL MeOH followed by 3 mL of nanopure water before samples were drawn through the system via vacuum pull. Following manufacturer instructions, the flow rate did not exceed 5 mL/min and atmospheres for the system did not exceed 20 Hg. After the entire 1-L sample volume passed through the column, it was flushed with 3 mL nanopure water. Elution was carried out by two successive additions of 2 mL MeOH to each column. Eluted samples were collected into 13 × 100-mm glass tubes and dried overnight under a stream of air, reconstituted in 500 μL assay buffer (0.045 M NaH_2_PO4, 0.061 M Na_2_HPO_4_, 0.149 M NaCl and 0.1% bovine serum albumin, pH 7.5), vortexed (30 s) and sonicated (5 min; Branson Ultrasonic Corp., Danbury, CT, USA) before being stored at −20°C until analysis.

### Enzyme immunoassay

Concentrations of immunoreactive corticosterone metabolites in faecal and water samples were quantified by enzyme immunoassay (EIA) employing a horseradish peroxidase (HRP)-conjugated label (1:100 000 dilution) and a polyclonal primary antibody (CMJ006; 1:80 000 dilution) provided by Coralie Munro, University of California, Davis. The primary antiserum cross-reacted with corticosterone (100%), desoxycorticosterone (14.25%) and progesterone (2.65%). Pre-coated secondary antibody-coated plates were utilized for the corticosterone EIA. The 96-well microtitre plates (Nunc-Immuno MaxiSorp, Fisher Scientific, Pittsburgh, PA) were prepared by adding a 150-μL volume of pre-diluted (10 μg/mL) goat anti-rabbit IgG (A009; Arbor Assays, Ann Arbor, MI, USA) in a coating buffer (X108, 20X; Arbor Assays) to all wells and incubating at RT for 24 h. Contents of each well were emptied, plates blotted dry and blocking solution (X109, 10X; Arbor Assays) added to each well (250 μL) before incubating again at RT for 24 h. After incubation, the contents of all wells were emptied, and plates were blotted dry and stored (RT, humidity ≤ 20%) until use in a Dry Keeper (Sanplatec Corp., Osaka, Japan) containing desiccant pellets. Before use, plates were removed from the Dry Keeper and kept at RT for a minimum 20 min before the assay buffer (0.045 M NaH_2_PO4, 0.061 M Na_2_HPO_4_, 0.149 M NaCl and 0.1% bovine serum albumin, pH 7.5) was added to the blank (100-μL) and zero (50-μL) wells. Corticosterone standards (range 78–20 000 pg/mL; E1752; Sigma-Aldrich), samples (50 μL) and internal controls (50 μL) diluted in the assay buffer were added in duplicate, combined with corticosterone–HRP (50 μL) followed by the addition of 50 μL primary antibody, except for blank wells, and shaken covered from light at RT for 2 h. Plates were washed three times with wash buffer before 100 μL of substrate solution (0.05 M C_6_H_8_O_7_, 0.4 mM ABTS) was added. After incubation for 30–60 min at RT with shaking, the absorbance was measured at 405 nM (VersaMax plate reader, Molecular Devices, Sunnyvale, CA, USA) once the optical density approached 0.9–1.0. Any results with ≥ 10% CV between duplicates were reanalyzed.

If sample results indicated binding was not within the linear portion of the standard curve, samples were diluted accordingly. In general, faecal extracts were run at a 1:2 or 1:3 dilution and extracted water samples were run neat except for the 4-hACTH challenge sample (1:10) due to its concentration exceeding the sensitivity of the assay. Internal controls (representing 78% and 44% binding or 2000 and 400 pg/mL, respectively) were added to each plate. Mean inter- and intra-assay variations for the corticosterone EIAs were <15%.

### Assay validation

The corticosterone EIA was validated for *N. beyeri* faecal extracts by conducting tests of parallelism, accuracy/recovery and extraction efficiency (Brown *et al*., 2005). To test for parallelism, pooled faecal extracts were serially diluted (neat-1:128) and assayed. Assay accuracy/recovery check was determined by spiking each standard 1:1 with pooled *N. beyeri* faecal extract containing a relatively low endogenous concentration (120 pg/well). Faecal extraction efficiency was determined by adding a known amount of standard (4000 pg/mL) to a pooled dried faecal sample prior to extraction. Similarly, efficiency of waterborne hormone extraction was analyzed through recovery of native hormone added to 1 L adjusted RO water (0.2 g MgSO_4_/L). Given that all waterborne hormone samples used the same onsite water source, the background *w*CM concentration was determined by extracting 1 L adjusted RO water that did not contain any exogenous hormone added to it.

**Figure 2:**
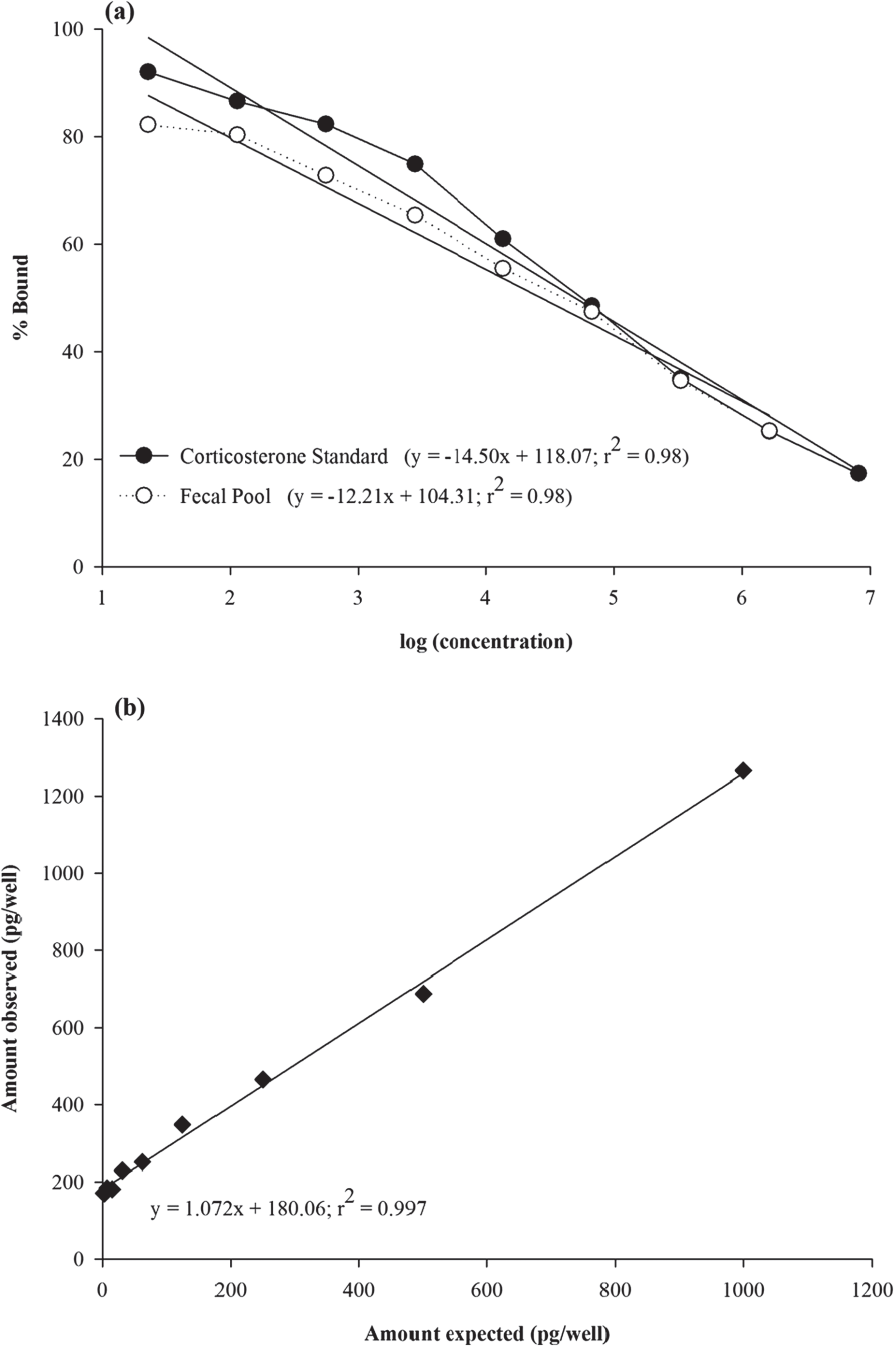
(**a**) Parallelism results for corticosterone in *Necturus beyeri* fecal samples; serial dilutions of pooled fecal extracts (open circles) are parallel to the standard (black circles) curve. Linear regression equations are given at the bottom. (**b**) Accuracy results for corticosterone in *N. beyeri* fecal extracts. Pooled feces spiked with known amounts of corticosterone showed good accuracy and little to no matrix effect. Regression equation given at bottom

**Figure 3:**
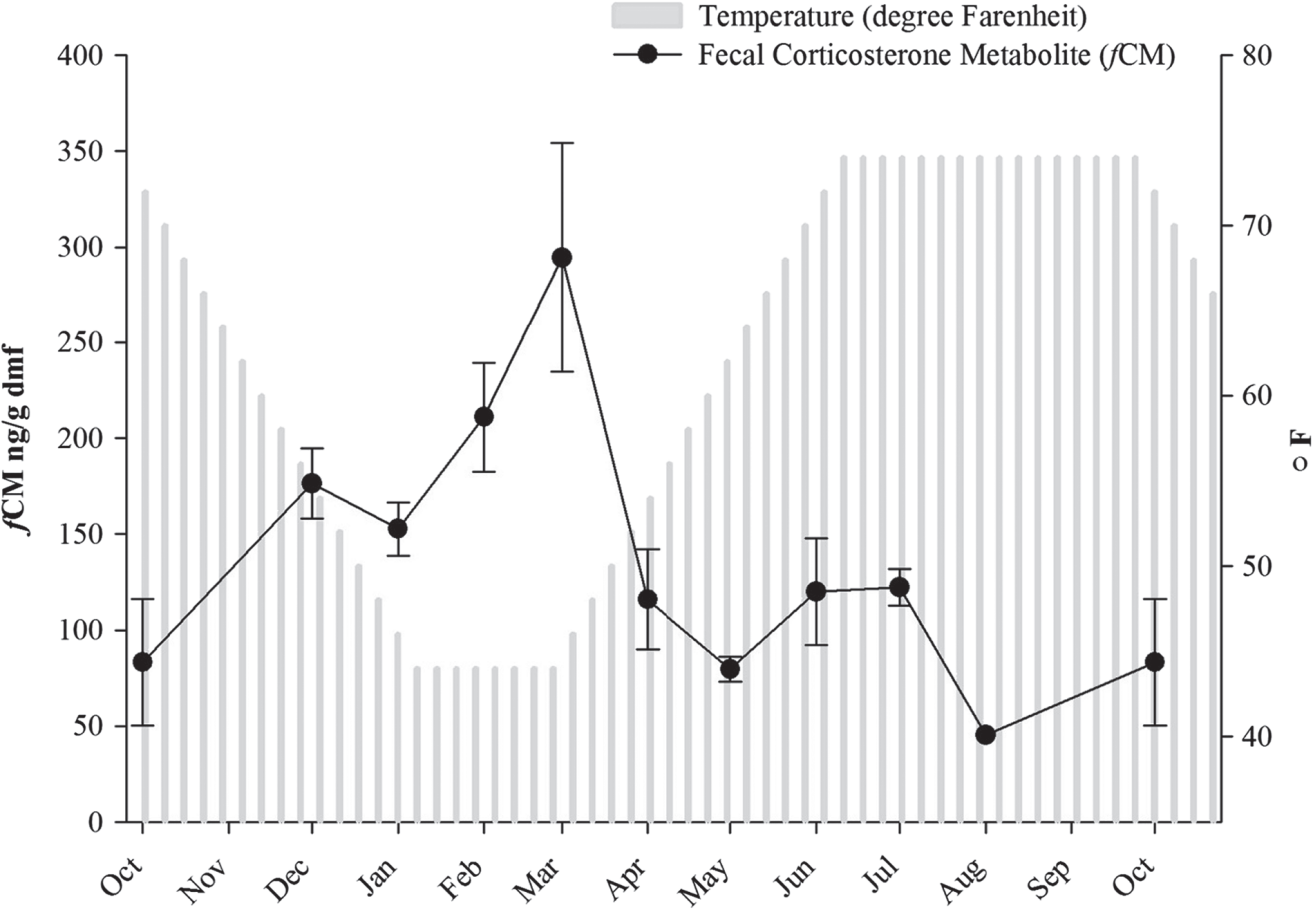
Weekly water temperature (°F) and mean ± SEM monthly fecal corticosterone (*f*CM) concentrations in 2016–2017 for six natural breeding pairs of *Necturus beyeri* at Jacksonville Zoo and Gardens. Fecal collection began in December 2016 and concluded in October 2017; data are presented with October 2017 adjacent to November 2016 for visual comparison

**Figure 4:**
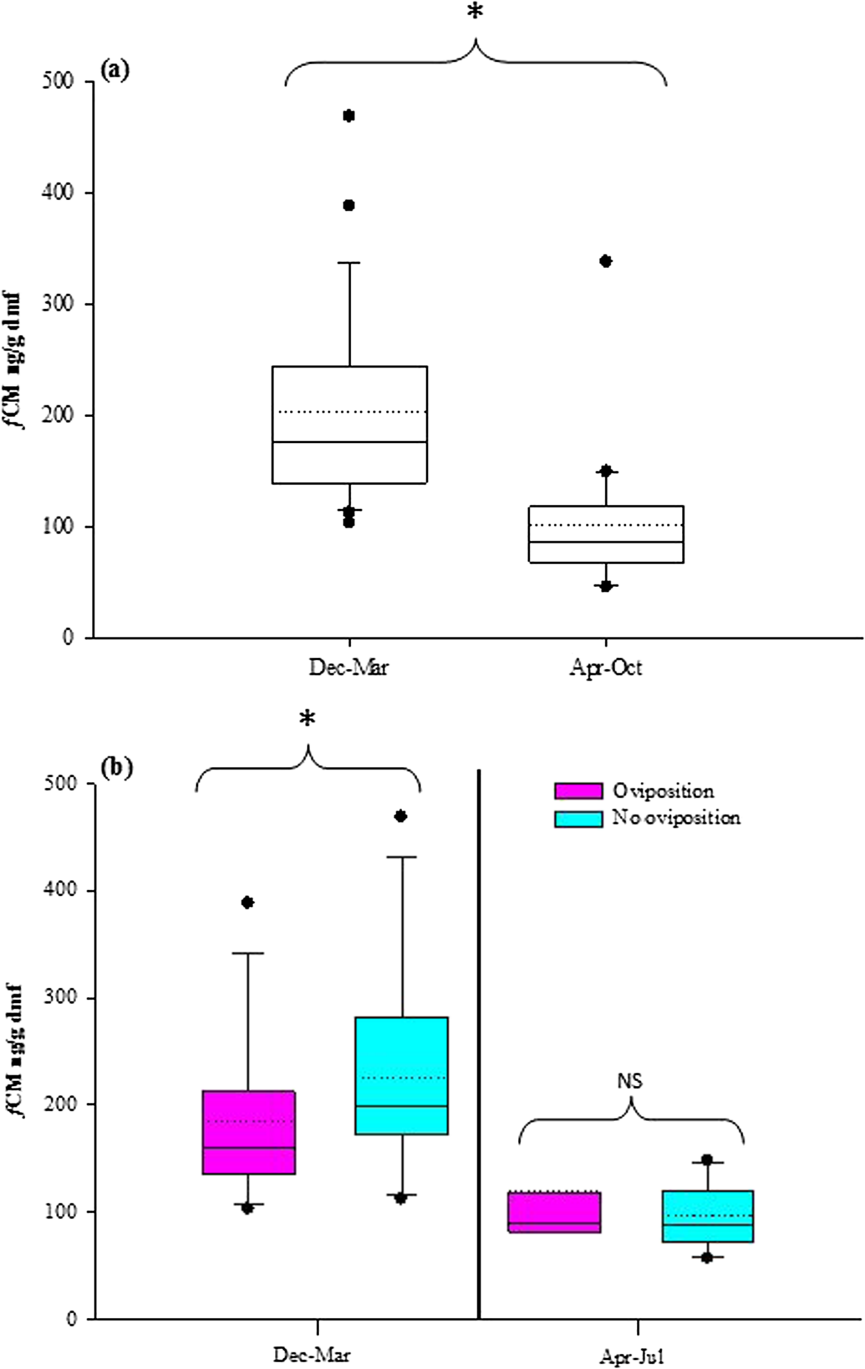
Concentrations of *f*CMs excreted between December–March and April–July of 2016–2017 from (**a**) captive *Necturus beyeri* breeding pairs (*n* = 6) and (**b**) pairwise comparison between breeding pairs whereby females went on to successfully oviposit eggs (*n* = 4) versus those that did not (*n* = 2). Box limits represent the 25th and 75th percentiles. Horizontal lines through the box represent the median values, dotted lines the mean value and outliers as black circles. Significance level between groups is indicated by * (*P* < 0.05), while non-significance (*P* > 0.05) is indicated by NS

**Figure 5:**
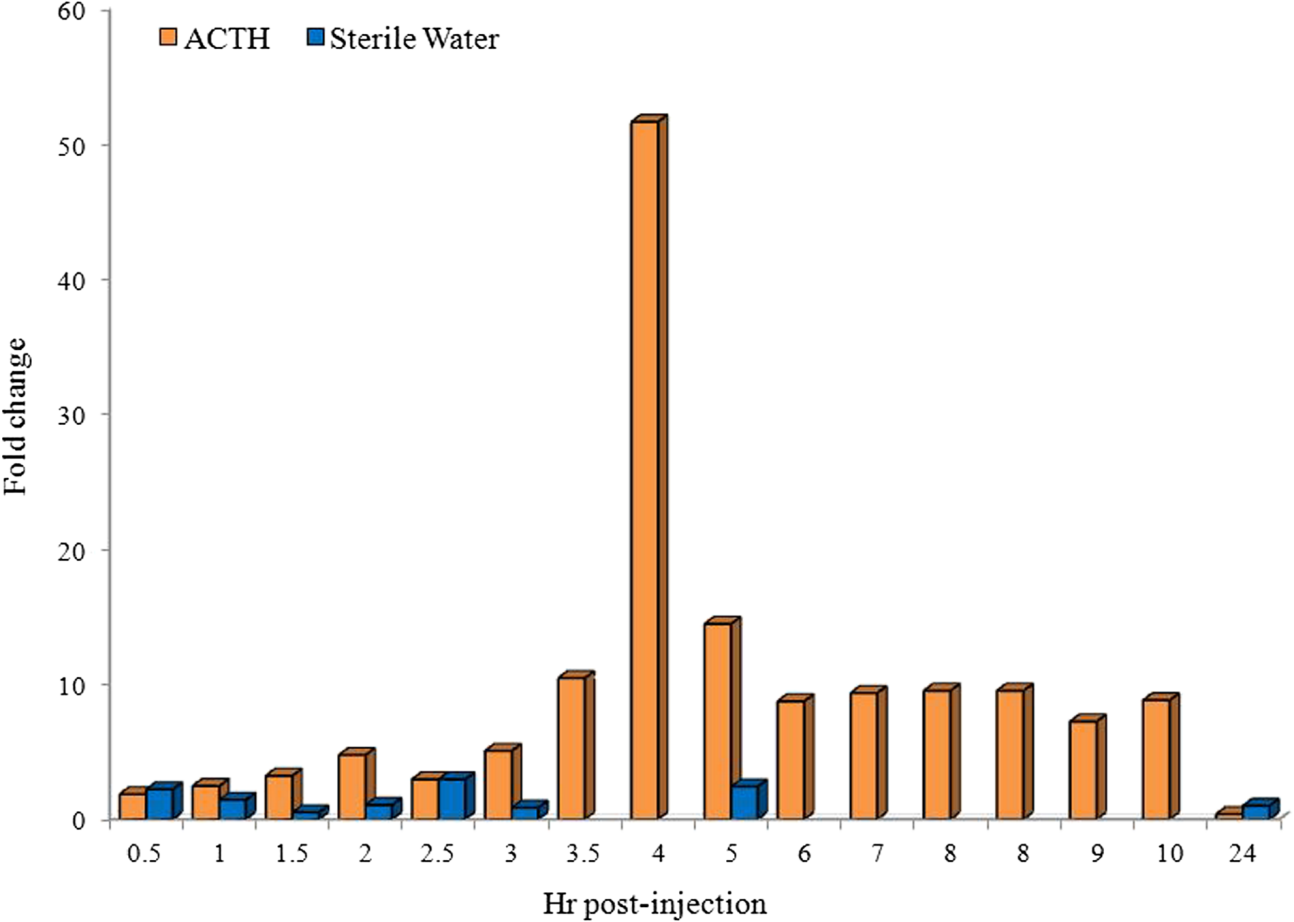
Fold change in waterborne corticosterone metabolite (*w*CM) concentrations of captive *Necturus maculosus* following anesthetic events associated with ACTH (*n* = 2) or sterile water (*n* = 1) administration

### Statistical analysis

Microsoft Excel and SigmaStat were used for statistical analysis. Standard descriptive statistics were used to summarize results. Data are presented as means ± SEM, and differences were considered significant at *P* < 0.05. Hormone data were normalized using logarithmic transformations. *T* tests were used to compare *f*CM concentrations between breeding (Dec–Mar) and non-breeding (Apr–Jul) months and *f*CM concentrations excreted between those breeding pairs where females did and did not oviposit eggs. Parallelism results for corticosterone were plotted as percentage of antibody bound versus log [relative dose]. Differences between slopes of the binding curves for the serially diluted pool and the standard curve were assessed with an *F* test. Accuracy/recovery check results were plotted as amount observed versus amount expected and assessed by linear regression, with acceptable accuracy defined as *r*^2^ ≥ 0.99 and slope within 0.85–1.15 (Brown *et al*., 2005). Extraction efficiency was determined by (amount observed—background/amount expected−background)*100. Individual values for faecal samples are represented in nanograms per gram of dry mass faeces (dmf). Raw *w*CM data (i.e. pg/mL) was multiplied by resuspension volume (0.5 mL), and the value of the background *w*CM sample was subtracted from salamander *w*CM values to determine the amount released by the animal. Finally, we divided by SVL to present the release rate of *w*CM in units of picograms per SVL. Concentrations of *w*CM following ACTH and sterile water injections are presented as fold change relative to baseline, with a basal concentration equal to 1.

## Results

### fCM and wCM assay validation

Corticosterone assay of serially diluted *N. beyeri* faecal extract yielded a displacement curve parallel to the standard curve (Fig. 2a). There was no significant difference in slopes between corticosterone standards and serially diluted *N. beyeri* faecal extracts (*F*_(1, 17)_ = 0.00397; *P* = 0.950; Fig. 2a). Accuracy/recovery check was acceptable as indicated by a linear relationship between observed and expected hormone concentrations (*r*^2^ ≥ 0.99) and a slope (1.07) within the desired range (Fig. 2b). The extraction efficiency of corticosterone added to dried faeces was 95%. The background *w*CM concentration of 1 L adjusted RO water was 65.5 pg/mL. An extraction efficiency of 98.5% was achieved when a known corticosterone concentration was added to 1 L of adjusted RO water.

### Physiologic measures

Spermatophores (*n* = 30) were deposited by males from all breeding pairs (range 1–10 spermatophores/pair); 89% were deposited from 08 December 2016 to 27 March 2017 with none reported after 05 April 2017. Four of six females went on to oviposit eggs (13 April 2017 to 23 April 2017); one clutch was fertile. In total, 122 faecal samples were collected from the six pairs (*n* = 62, Dec–Mar; *n* = 46, Apr–Jul; *n* = 14, Aug–Oct). Peak *f*CM concentrations were excreted in March (294.39 ± 59.84 ng/g dmf) followed by a precipitous decline in April (115.99 ± 26.14 ng/g dmf; Fig. 3). Higher *f*CM concentrations (203.67 ± 17.96 dmf) were excreted during months associated with lower water temperatures and breeding (spermatophore deposition and egg growth) compared to non-breeding (egg-laying) activity (105.61 ± 12.51 ng/g dmf; *P* < 0.05; Fig 4a). There was no difference in *f*CM concentrations between breeding pairs that had females that went on to successfully oviposit eggs versus those that did not (*P* > 0.05; Fig. 4b).

### ACTH challenge

A 50-fold increase in *w*CM concentrations (baseline: 3.46 pg/SVL vs. peak: 178.73 pg/SVL) was observed 4 h following ACTH injection and remained at a ≥7-fold increase (range 7.23–14.46) from 5 to 10 h post-injection (Fig. 5). Maximal *w*CM concentrations measured in response to the anaesthetic event associated with sterile water injection (control) that occurred for 2.5 h (10.2 pg/SVL) post-recovery, resulting in a 2.9-fold change (Fig. 5). Concentrations of *w*CM returned to baseline 24 h after anaesthetic events associated with the injection of ACTH (1.29 pg/SVL) or saline (3.41 pg/SVL).

## Discussion

Innumerable threats to biodiversity exist, but none so much as for species inhabiting freshwater ecosystems like *Necturus*. Establishing endocrine correlates associated with *Necturus* reproductive dynamics serve to benefit conservation and research efforts aimed at promoting breeding in managed settings. Two practical non-invasive methods of faecal and waterborne hormone collection were validated for monitoring *Necturus* inter-renal activity. Alternate choices now exist to traditional blood or tissue sampling that can have confounding effects due to the capture, anaesthesia and restraint necessary to obtain samples (Hernandez *et al*., 2006). Longitudinal *f*CM measurement provided evidence to support a seasonal inter-renal effect in captive *Necturus*. As elevated plasma corticosterone concentrations have been shown to increase metabolism in terrestrial salamander species (*Plethodon shermani*, Wack *et al*., 2012), it seems plausible that the *f*CM changes observed in this study could be associated with the shifting energetic demands necessary to carry out the seasonal reproductive strategy of *Necturus*. While the terrestrial salamander *Ambystoma* fails to show a seasonal pattern in plasma corticosterone (Homan *et al*., 2003), crested newts exhibit two peaks in corticosterone: one at the start of reproduction in Winter and the second during Summer as they leave the pond (Zerani and Gobbetti, 1993). Similarly, the American toad exhibits a peak in corticosterone during the Spring and again in the Fall (Pancak and Taylor, 1983). In this study, *f*CM concentrations were excreted during the breeding season, a time of marked water temperature decline and when males from each breeding pair deposited spermatophores. This is also when *Necturus* egg development and growth take place (Calatayud *et al*., 2019, Stoops *et al*., 2014). A sharp decline in *f*CM in April was concomitant with warming water temperatures, a cessation of breeding activity and the oviposition of eggs by four of six paired females. A similar *f*CM decline was also noted in two breeding pairs where the female did not go on to oviposit eggs. Oviposition timing matched that previously observed in this and two other captive *Necturus* species (Calatayud *et al*., 2019, Stoops *et al*., 2014). As this study did not employ ultrasound technology, it remains unknown if the two females that failed to deposit eggs did so because of lack of egg development versus having matured eggs that failed to ovulate and/or oviposit (Calatayud *et al*., 2018, 2019).

Amphibian reproductive cycles are influenced by changes in environment (Belden *et al*., 2001). However, the degree to which a species relies on particular cues such as light, temperature and rainfall is highly specific (Todd *et al*., 2011). Sex of an individual can also influence the response to environmental change (Sexton *et al*., 1990). This initial study in *N. beyeri* correlated changes in water temperature to inter-renal and gonadal function. Fine-tuning the degree to which changes in water temperature drive reproductive physiology and behaviour in this species will be an important next step in improving captive breeding protocols. Preliminary evidence has emerged to show that a reduction in temperature differential between seasons significantly reduces *f*CM concentrations and reproductive events in *N. beyeri* (unpublished data). From a conservation perspective, environmental changes and the effects thereof on reproductive dynamics are important for understanding the potential ramifications of climate change on a given species.

While *f*CM data provided information regarding inter-renal activity as it relates to observed reproductive events and seasonal changes in water temperature experienced by captive *Necturus*, the specific sex of deposited faecal samples could not be differentiated within each breeding pair as males and females were housed together year-round. However, collection and analysis of communal faecal samples from individual species can serve as important bio-monitoring tools for population level assessments (Guertin *et al*., 2010). Corticosterone concentrations have been shown to differ between male and female terrestrial salamanders *Ambystoma*, but not year-round (Homan *et al*., 2003). Given the scope of our study, we could not substantiate whether inter-renal hormone production differences also exist between the *N. beyeri* sexes. Despite some limitations imposed by faecal hormone collection/analysis and given the improbability of obtaining faecal samples from wild specimens for comparison, we sought to validate a non-invasive waterborne hormone monitoring technique. As a result, the first waterborne corticosterone response to ACTH in *Necturus* was demonstrated with *w*CM concentrations significantly increasing 3.5–4 h post-injection. The ACTH challenge is common practice to physiologically validate adrenal hormone assays in mammalian, reptile and amphibian species (Capiro *et al*., 2014, Gendron *et al*., 1997, Graham *et al*., 2013, Narayan *et al*., 2010a, Santymire *et al*., 2018, Scheun *et al*., 2019). Other amphibian waterborne hormone studies only sampled measures over a 1-h(Gabor *et al*., 2016) or ~2-h(Reedy *et al*., 2014) timeframe post-ACTH with injection achieved via physical restraint. Similar to Gabor *et al.* (2016), we found no difference in *w*CM concentrations at 1 h when the male was injected with saline or sterile water compared to ACTH. In *Notophthalmus*, statistically significant differences in waterborne corticosterone were documented by ~2 h following ACTH administration (Reedy *et al*., 2014). As we did not observe a significant difference in *w*CM between our first ACTH challenge and sterile water control over a 3-h timeframe, a second ACTH challenge was conducted with *w*CM collection, extraction and assay analysis extended over 10 h. After an initial significant rise at 3.5 h and peak at 4 h, *w*CM concentrations remained >7-fold higher up to 10 h, with basal concentrations achieved by 24 h. Similar to Santymire *et al.* (2018) in which a 2-fold increase in dermal secretion of cortisol was measured from *N. maculosus* 2 h post-restraint stress, we observed a 2.9-fold increase in *w*CM at 2.5 h following the anaesthetic event associated sterile water i.p. injection. As this is the first study to validate waterborne hormone analysis in a large fully aquatic amphibian species, the volume of water for collection was increased 10 to 25 times that reported for *Eurycea* (Gabor *et al*., 2013, 2016), *Alytes* (Gabor *et al*., 2013) and *Notophthalmus* (Reedy *et al*., 2014). However, the time at which *Necturus* remained in water for collection of hormone was consistent with previous reports (Gabor *et al*., 2013, 2016, Reedy *et al*., 2014). Background *w*CM concentrations reported in this study were similar to those reported by Reedy *et al*. (2014).

This is the first study to validate faecal hormone analysis in a salamander species with important insight gained into inter-renal gland function as it relates to the seasonal reproductive strategy employed by this cryptic amphibian. As *Necturus* appear susceptible to the reproductive challenges faced by other amphibian captive propagation programs (i.e. low fertility), developing effective means to measure inter-renal and gonadal activity will aid in determining whether specific changes in husbandry, management or assisted breeding protocols have positive or negative impacts on reproduction. In addition, it should provide a greater understanding of *Necturus* physiological function including stress responses, which is especially important as human disturbance and climate change may lead to the decline of wild populations.
